# Quantifying the depth of anesthesia based on brain activity signal modeling

**DOI:** 10.1097/MD.0000000000018441

**Published:** 2020-01-31

**Authors:** Hyub Huh, Sang-Hyun Park, Joon Ho Yu, Jisu Hong, Mee Ju Lee, Jang Eun Cho, Choon Hak Lim, Hye Won Lee, Jun Beom Kim, Kyung-Sook Yang, Seung Zhoo Yoon

**Affiliations:** a Department of Anesthesiology and Pain Medicine, Anam Hospital, Korea University College of Medicine; b Medical Device Innovation Center, Korea University Medical Center; c KM Fundamental Research Division, Korea Institute of Oriental Medicine, Daejeon; d Department of Biostatistics, Korea University College of Medicine, Seoul, Republic of Korea.

**Keywords:** anesthesia, anesthetic depth, bispectral index, cortical activity index, electroencephalogram, spectral entropy

## Abstract

Various methods of assessing the depth of anesthesia (DoA) and reducing intraoperative awareness during general anesthesia have been extensively studied in anesthesiology. However, most of the DoA monitors do not include brain activity signal modeling. Here, we propose a new algorithm termed the cortical activity index (CAI) based on the brain activity signals. In this study, we enrolled 32 patients who underwent laparoscopic cholecystectomy. Raw electroencephalography (EEG) signals were acquired at a sampling rate of 128 Hz using BIS-VISTA^TM^ with standard bispectral index (BIS) sensors. All data were stored on a computer for further analysis. The similarities and difference among spectral entropy, the BIS, and CAI were analyzed. Pearson correlation coefficient between the BIS and CAI was 0.825. The result of fitting the semiparametric regression models is the method CAI estimate (−0.00995; *P* = .0341). It is the estimated difference in the mean of the dependent variable between method BIS and CAI. The CAI algorithm, a simple and intuitive algorithm based on brain activity signal modeling, suggests an intrinsic relationship between the DoA and the EEG waveform. We suggest that the CAI algorithm might be used to quantify the DoA.

## 1 Introduction

Assessing anesthetic depth and reducing intraoperative awareness during general anesthesia has historically been of great interest in the field of anesthesiology.^[1]^ Even though 10 million patients undergo surgery under general anesthesia every year,^[2]^ 1 in every 1000 to 2000 may temporarily regain consciousness or even remain conscious during surgery.^[3]^ Awareness during surgery is associated with severe psychological sequelae such as posttraumatic stress disorder.^[3]^ Consequently, quantifying both the depth of anesthesia (DoA) and the level of consciousness of patients is a very important research area in anesthesiology.

Many scientists have attempted to know how general anesthetics induce unconsciousness, amnesia, and immobility, but the mechanism is not completely understood^[4]^; however, it is known that anesthetics suppress cortical neuronal activity. Thus, analysis of the change in electroencephalography (EEG) pattern in relation to various factors such as anesthetic agents, methods of anesthesia, and patient illnesses is a popular research topic.^[5,6]^ A few DoA systems that quantify brain activity have been well developed with advanced mathematical algorithms based on a large database of human studies. The bispectral index (BIS, Covidien, Norwood, MA) and spectral entropy (SpEn; E-Entropy, GE Healthcare, Helsinki, Finland) are well-known examples of DoA monitors utilizing EEG signals.^[7]^ BIS and SpEn are commonly used to guide the administration of volatile and intravenous (IV) anesthetics as an index of the DoA.^[8]^ The BIS algorithm is a weighted summation of 3 processed variables: beta ratio, synch fast slow, and burst suppression ratio.^[5,7]^ BIS is based on the bispectral analysis of EEG, which is modified based on statistics derived from a large human database, combining the 3 aforementioned subparameters.^[9,10]^ The usefulness of BIS in monitoring anesthesia in patients has been well established.^[11,12]^ Meanwhile, the SpEn algorithm uses irregularity in the EEG and frontal electromyogram power spectrums to calculate 2 indexes: state entropy and response entropy.^[7]^

However, there are limitations for both BIS and SpEn algorithms; for example, these monitors fail to accurately represent DoA with regard to certain factors such as the type of anesthetic agent and patient characteristics.^[13]^ One of the main reasons for the failure is the lack of signal modeling to quantitatively describe the change in EEG during anesthesia. Understanding the mechanism of the generation of EEG has to precede the development of the signal model. The main sources of EEG are the postsynaptic potentials from the dendrites of the pyramidal cells in the cortical layer.^[10,14]^ These signals summate and are recorded as EEG activity at the surface of skin. In other words, EEG represents the summated electrical activity of multiple sources located in the cortical layer, with the most significant contribution from the superficial cortical layers. During wakefulness, excitatory input from other neurons provides a depolarizing drive that cause neurons to exhibit single-spike tonic firing. During anesthesia, neurons switch into a burst-firing mode. At intermediate anesthetic concentrations, neurons oscillate between an active up-state and an inactive down-state. As anesthetic dose increases, the up-state turns into a short burst and the down-state becomes progressively longer. Therefore, the percent of pyramidal cells in the active state can be considered as a parameter that is directly reflecting the DoA.

We hypothesize that a signal model that gives a quantitative description of changes in EEG with respect to the change in the percentage of active pyramidal cells can explain not only many features of EEG during anesthesia, such as burst suppression and a decrease in high-frequency component, but also changes in the indexes for the DoA. In addition, based on the signal modeling, a time-domain method can be proposed to estimate the DoA from EEG segments. Therefore, our primary end point was to validate the proposed algorithm (cortical activity index [CAI]), by using it to measure EEG signals from anesthetized humans and estimate the DoA.

## 2 Materials and methods

### 2.1 Brain activity signal modeling: CAI

If a brain cell is activated by an external stimulus that exceeds a particular threshold, the cell generates an electrical peak signal that lasts for a short time with a relatively large amplitude. While a patient is awakening, the brain activity is comparatively high, and it can increase the probability that the brain cell produces peak signals. Since an EEG recording is an aggregation of peak signals generated from the brain cells, the EEG signal in the awakening state has more peak signal components. This is related to the features of the EEG signal. As anesthesia progresses, the frequency components of the EEG signal change. Furthermore, the reason for this change in pattern could be related to the fact that the ratio of peak signals contained in the EEG signal is rather low when a patient is in a hypnotic state.

The fundamental process of CAI is to calculate the ratio of peak signals present in the observed EEG signal.^[15]^ According to our definition, a peak signal is a portion of the observed EEG signal that is higher than a predefined threshold in its amplitude. Following this definition, we can calculate the density of the peak signals in every epoch to derive CAI. Here, the density of the peak signal is represented by the number of peak signal points per epoch divided by all the signal points contained in 1 epoch. This ratio is directly correlated with CAI. A high ratio indicates an awakening state, and a low rate represents a deep hypnotic state. At this point, the threshold that is applied to each epoch in the time domain should be adaptive because the magnitude of the EEG signals change during a surgical procedure. If the threshold is fixed during the surgery, the derived value for the DoA will not be appropriate. Since the amplitude of an EEG signal is low in an awakening state, a low threshold has to be used. On the other hand, a high threshold is applied during deep hypnotic state. The amount of low-frequency components in the EEG signal is high when a patient is anesthetized. Hence, using this feature, the adaptive threshold can be defined as follows in eq. 1.^[15]^ In eq. 1, *X* represents an EEG signal, DFT denotes Discrete Fourier Transform, and *K* and *M* are experimentally derived constant values. In other words, this threshold quantifies the low-frequency components of the observed signal. In summary, we can obtain CAI by calculating the adaptive thresholds initially and quantifying the density of the peak signal components that are above the adaptive thresholds in each epoch^[15]^: 
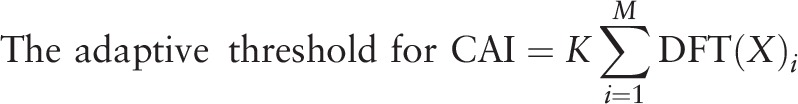


### 2.2 Human anesthesia and data acquisition

This study was approved by the institutional review board of Korea University Medical Center (MD15010) and is registered at ClinicalTrials.gov (NCT02586441). After obtaining written informed consent from each patient, 32 patients (American Society of Anesthesiologists physical status I or II, age >18 years) scheduled to undergo laparoscopic cholecystectomy under general anesthesia were included in this study. Exclusion criteria were medical conditions that might affect the level of consciousness such as stroke, altered mental state, or dementia.

The patients were administered with glycopyrrolate 0.2 mg as premedication intramuscularly 30 minutes before anesthesia. Standard monitoring included electrocardiography, noninvasive arterial blood pressure, pulse oximetry, and the BIS-VISTA^TM^ sensor. Thereafter, anesthesia was induced with IV 1% propofol (1.5–2.5 mg kg^−1^) and 0.6 mg kg^−1^ rocuronium bromide (Esmeron; Merck Sharp and Dohme, Oss, Netherlands) to facilitate tracheal intubation, followed by initiation of mechanical ventilation. Tidal volume and respiratory rate were set at 8 to10 mL kg^−1^ and 10 to 12 per minutes, respectively. During the surgery, respiratory rate was adjusted to achieve normocarbia. Anesthesia was maintained with desflurane at an end-tidal concentration of 6% to 7%, with a fraction of inspired oxygen of 0.5 (fresh gas flow; O_2_ at 1.5 L/min and air at 2.5 L/min). Postoperatively, anesthesia was discontinued and the fractional inspired oxygen concentration was increased to 1.0. The tracheal tube was removed when the patient demonstrated purposeful movement and facial grimacing and was breathing spontaneously and regularly. After extubation, BIS-VISTA^TM^ monitoring was stopped, and the patients were transferred to a post-anesthesia care unit. Raw EEG signals were acquired at a sampling rate of 128 Hz using BIS-VISTA^TM^ with standard BIS sensors. All data were stored on a computer for further analysis.

### 2.3 Statistical analysis

We obtained Pearson correlation coefficients among the 3 indexes (the CAI, SpEn, and BIS). Owing to the disparate ranges for each index, CAI and SpEn were normalized using the following formula: 
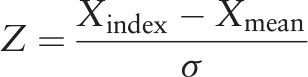


for the agreement of indexes or fitting statistical models, where *Z* is the standardized index, *X* is CAI and SpEn, and *σ* is the standard deviation for each index for statistical analysis.

We fitted the semiparametric regression model to test the differences among the ZCAI, ZSpEn, and ZBIS after adjusting for the effect of sex, age, body mass index (BMI), as well as the linear and nonlinear effect of time. We additionally fitted the semiparametric regression model by each index. Semiparametric regression models are regression models that include both parametric and nonparametric regression models. In the nonparametric regression model, a smoother is used for fitting the trend of a dependent variable as a function of one or more independent variables. Here, the nonparametric model is expressed with smooth functions. When the smoothing parameter is close to 1, a smoother curve is generated. Conversely, when it is close to 0, a rougher curve is generated. The value of the smoothing parameter was selected by generalized cross-validation.

All statistical analyses were performed using IBM SPSS Statistics version 22.0 (IBM Corporation, Armonk, NY), R 3.1.3 (The R Foundation for Statistical Computing, Vienna, Austria), and SAS 9.4 (SAS Institute Inc, Cary, NC). We considered *P* < .05 as statistically significant.

## 3 Results

### 3.1 Similarity between the CAI and BIS

**Figure 1:**
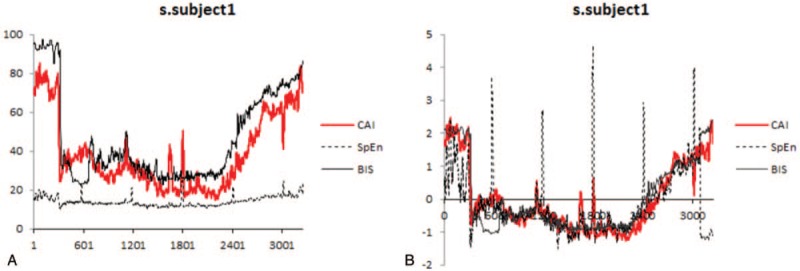
A typical graph of human data showing CAI, SpEn, and BIS during the surgery; (A) shows the absolute CAI, SpEn, and BIS. (B) shows their normalized index with standardization. BIS = bispectral index, CAI = cortical activity index, SpEn = spectral entropy.

**Figure 2:**
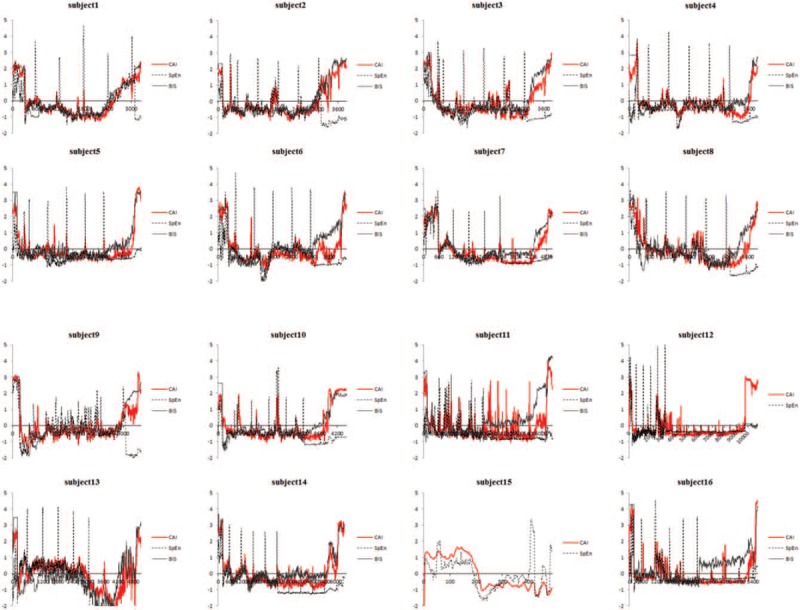
The standardized CAI (ZCAI), SpEn (ZSpEn), and BIS (ZBIS) for human data are presented for each subject. BIS = bispectral index, CAI = cortical activity index, SpEn = spectral entropy.

Patients’ characteristics are presented in Table 1. The typical graph of the CAI, SpEn, and BIS during the complete anesthetic period is presented in Figure 1. The graphs of the ZCAI, ZSpEn, and ZBIS for all the human clinical trials are presented in Figure 2. BIS was not measured in 1 subject (subject 15). Pearson correlation coefficients were 0.803 between the CAI and SpEn; 0.617 between the SpEn and BIS; and 0.825 between the CAI and BIS (Table 2), respectively. CAI strongly correlated (>0.8) with both BIS and SpEn, but the correlation of SpEn with BIS was lower.

**Table 1:**
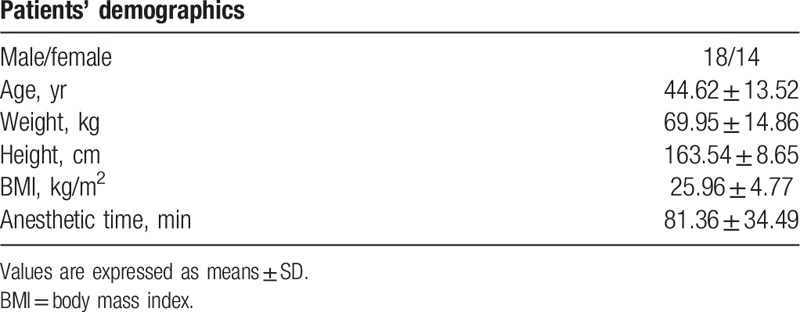
Patients’ characteristics.

**Table 2:**
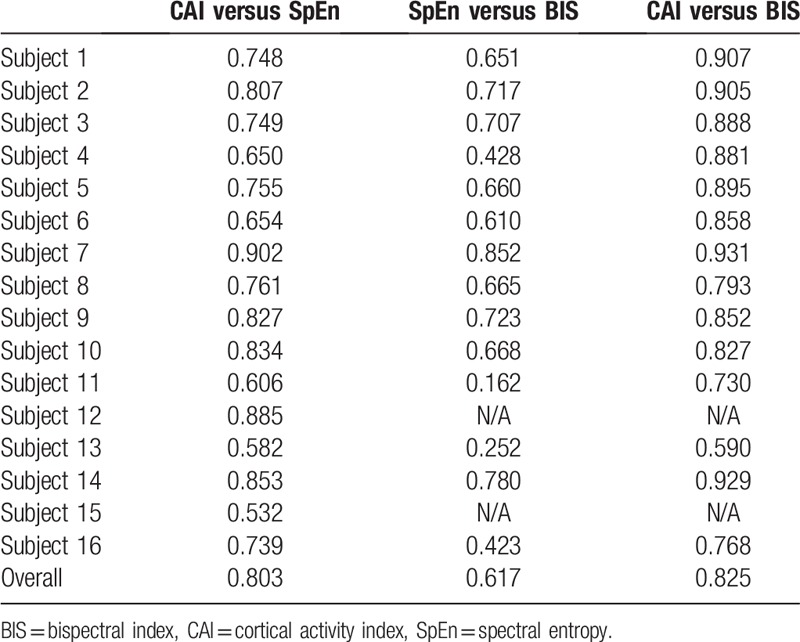
Pearson correlation coefficient of CAI versus SpEn, SpEn versus BIS, and CAI versus BIS in human subjects.

### 3.2 Differences between the CAI and BIS

In the basic statistical analysis, the minimum values were −1.3, −1.5, and −10; the median values were −0.33, −0.4, and −0.5; and the maximum values were 2.5, 4.7, and 2.5 for the ZCAI, SpEn, and BIS, respectively (Table 3). The absolute values of |Max–Min| were 3.8, 6.2, and 3.5 for ZCAI, ZSpEn, and ZBIS, respectively. The result of fitting the semiparametric regression model (except for subject 15) revealed the difference among the 3 algorithm performances along the time domain (Table 4).

**Table 3:**
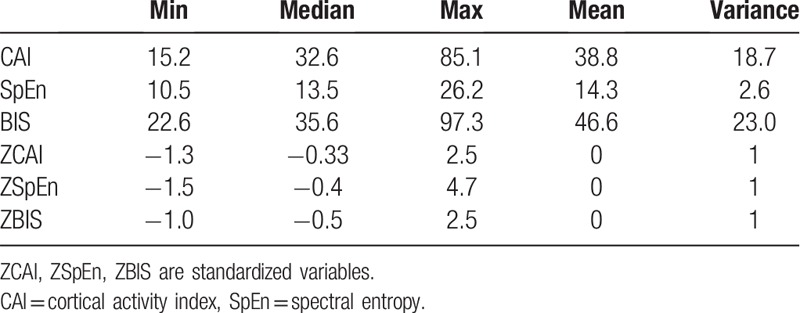
Descriptive statistics of the CAI, SpEn, and BIS for the human dataset.

**Table 4:**
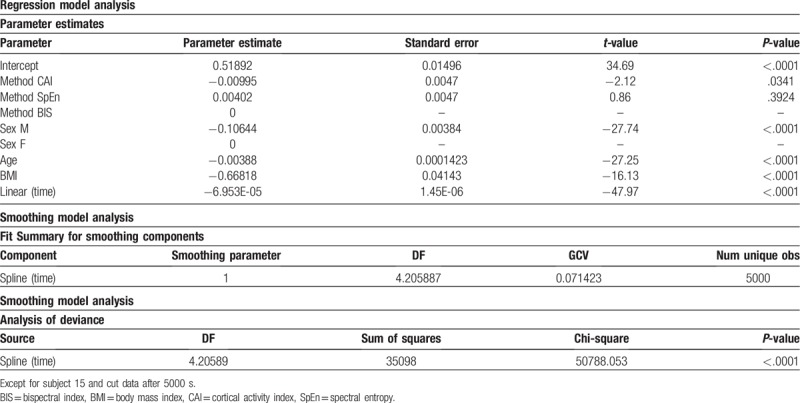
The results of fitting the semiparametric regression model (except for subject 15, cutting data after 5000 s) for the human dataset.

**Figure 3:**
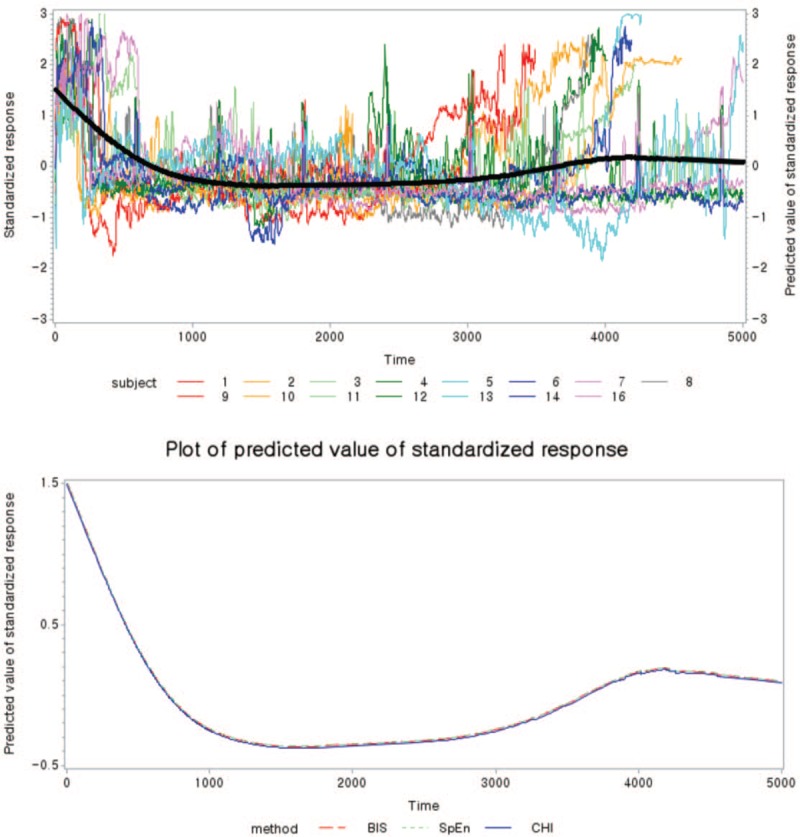
(A) The fitted (denoted by bold lines) plot from the semiparametric regression model. (B) The fitted (denoted by bold lines) plot from the semiparametric regression model by the CAI, SpEn, and BIS. BIS = bispectral index, CAI = cortical activity index, SpEn = spectral entropy.

In Table 4, the “Method” CAI estimate (−0.00995; *P* = .034) is the estimated difference in the mean of the dependent variable between “Method” CAI and BIS, after adjusting for the effect of the other variables in the model. The “Method” SpEn estimate (0.00402; *P* = .39) is the estimated difference in the mean of the dependent variable between “Method” SpEn and BIS, after adjusting for the effect of the other variables in the model. There was no significant difference in the “Method” between SpEn and the BIS. The “male sex” estimate (−0.10644; *P* < .001) is the estimated difference in the mean of the dependent variables between male and female sexes, after adjusting for the effect of the other variables in the model. The reference group is Female. The “Age” estimate (−0.00388; *P* < .001) is the estimated slope for the BIS of “Method” in the Female group, after adjusting for the other effects. Owing to the negative slope, the dependent variable decreases as “Age” increases. The “BMI” estimate (−0.66818; *P* < .001) is the estimated slope for the BIS of “Method” in the Female group after adjusting for other effects. Due to the negative slope, the dependent variable decreases as “BMI” increases. The “Time” estimate (−6.953 × 10^−5^; *P* < .001) is the estimated slope for the BIS of “Method” in the Female group, after adjusting for the remaining effects. On account of the negative slope, the predicted value of the dependent variable decreases as “Time” increases. In Table 4, the nonlinear contribution of the variable “Time” in the nonparametric model using the spline function yields significant results in the smoothing model analysis. When the smoothing parameter is close to 1, and we could obtain a smooth curve. From this result, the effect of CAI is significantly different from that of the BIS; however, the effect of SpEn is not significantly different from that of the BIS, after adjusting for the effect of the remaining variables such as sex, age, BMI, and time. It indicates that the overall trend of the data over time was similar; however, it showed a significant difference between the CAI and BIS methods, as shown in Table 4 and Figure 3.

**Figure 4:**
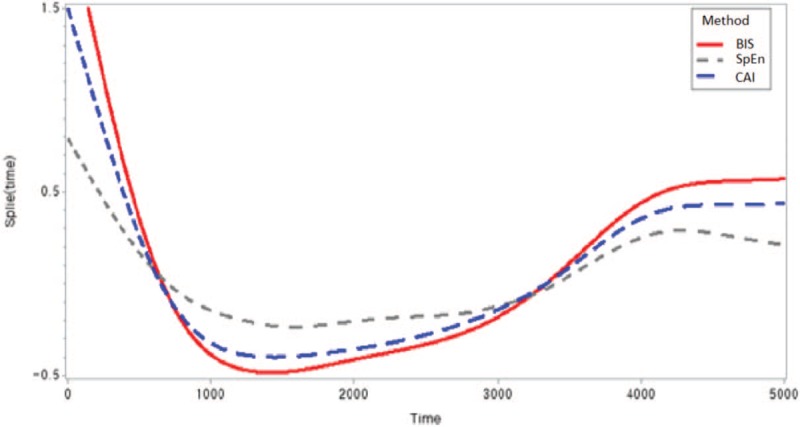
The effect of nonparametric components plot from the semiparametric regression model for each index.

Table 5 shows the results of fitting the semiparametric regression models for each BIS, SpEn, and CAI, respectively. For the CAI, the “Method” CAI estimate (−0.00995; *P* = .03) is the estimated difference in the mean of the dependent variable between “Method” CAI and BIS, after adjusting for the effect of the remaining variables in the model. For the CAI, the estimated parameters of sex, age, BMI, and the linear time effect were −0.09651, 0.00365, 0.51868, and −0.0001257, respectively, at the baseline of the female group (*P* < .001); for SpEn, the values were 0.0805, 0.00445, 0.38957, and 0.0000306, respectively (*P* < .001); and for the BIS, the values were 0.14748, 0.00351, 1.1332, and −0.000005003, respectively (*P* < .001). Figure 4 shows the smoothing component plot for each BIS, SpEn, and CAI. Nonparametric prediction for all 3 variables was significant. The CAI showed lesser difference than did BIS for sex and BMI, and it decreased faster compared with the BIS and SpEn for the linear time effect.

**Table 5:**
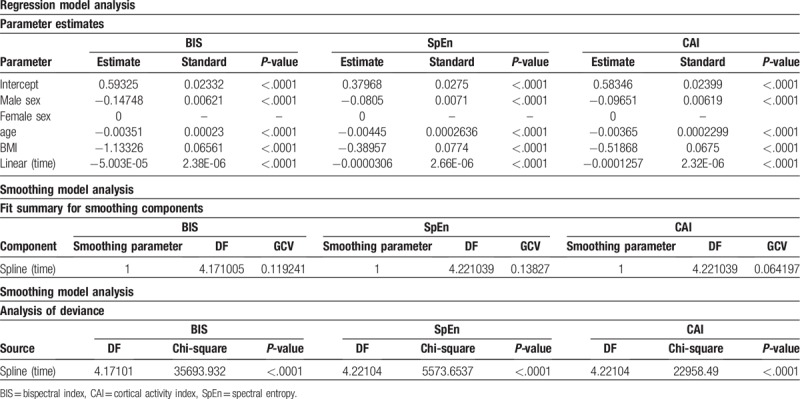
The results of fitting the semiparametric regression model by each index: CAI, SpEn, and BIS in the human dataset.

## 4 Discussion

In this study, we propose a new algorithm termed “CAI” based on brain activity signal modeling with various regression models. Our results showed that the proposed CAI algorithm was highly consistent and strongly correlated with the results from BIS in humans.

Based on the possibility that the CAI algorithm may predict the DoA, an experiment using human EEG was designed with 3 algorithms: CAI, SpEn, and BIS. After a standardization process, the Pearson correlation coefficient between CAI and BIS showed that correlation between CAI and BIS was the highest. Many previous clinical studies have shown that BIS can provide appropriate index values of the DoA in a steady state.^[16–18]^ Therefore, the authors suggest that a high correlation between BIS and CAI may indicate that the CAI algorithm can predict DoA reliably. Interestingly, according to the semi-parametric regression model, the CAI values were significantly different from the BIS values with regard to sex, age, BMI, and time values for overall time (*P* < .001) (Table 4). Thus, the authors hypothesize that the CAI may have a faster response time in representing recent brain activity than the BIS (−6.953E-05, *P* << .001)

According to the Pearson correlation coefficients, CAI was strongly correlated (>0.8) with both BIS and SpEn, but SpEn showed a lower correlation with BIS. In addition, the descriptive statistics of CAI, SpEn, and BIS revealed that SpEn was significantly lower than CAI or BIS. However, the descriptive statistics of ZSpEn were not different from those of ZCAI or ZBIS. Moreover, according to the semiparametric regression model, there was no significant difference for the “Method” between SpEn and the BIS. The “Method” SpEn estimate (0.00402; *P* = .39) is the estimated difference in the mean of the dependent variable between “Method” SpEn and BIS after adjusting for the effect of the other variables in the model. Thus, the authors conclude that there were no statistical differences between CAI, BIS, and SpEn.

There are some limitations to our research. The first is that the number of patients enrolled in the present study is very small. The second is that we may not have clearly interpreted statistically and clinically significant data. Finally, the novel DoA measurement method proposed in the current study does not provide any advantages over the existing methods. Therefore, it is likely that subsequent large clinical trials will be required. In future studies, a careful study protocol that takes into account the unanswered questions in this study is needed.

In this study, we focused on brain electrical activity, notably cortical neuronal activity. Furthermore, we hypothesized that the percentage of pyramidal cells in an active state can be considered a parameter that directly reflects the DoA. Based on the brain activity signal modeling that estimates the changes in EEG with respect to the proportion of active pyramidal cells, the CAI algorithm was developed with the dynamic threshold decision method. The proposed CAI algorithm is a simple and intuitive one based on brain activity signal modeling that suggests an intrinsic relationship between DoA and EEG waveform. Furthermore, the CAI algorithm might prove useful and more accurate in predicting the level of consciousness compared with other algorithms. In summary, we propose that the newly formed CAI algorithm is comparable to the well-established algorithms used in current DoA monitors, and it can be considered a suitable alternative for future DoA monitoring systems.

## Author contributions

**Conceptualization:** Hyub Huh, Sang-Hyun Park, Seung Zhoo Yoon.

**Data curation:** Sang-Hyun Park, Joon Ho Yu, Jisu Hong, Mee Ju Lee, Jang Eun Cho, Choon Hak Lim, Hye Won Lee.

**Formal analysis:** Sang-Hyun Park, Jun Beom Kim, Kyung-Sook Yang.

**Funding acquisition:** Hyub Huh.

**Investigation:** Hyub Huh, Jisu Hong, Mee Ju Lee, Jang Eun Cho, Hye Won Lee.

**Methodology:** Hyub Huh, Sang-Hyun Park, Choon Hak Lim, Hye Won Lee, Jun Beom Kim.

**Project administration:** Hyub Huh, Sang-Hyun Park.

**Resources:** Jun Beom Kim, Kyung-Sook Yang.

**Software:** Jun Beom Kim.

**Supervision:** Seung Zhoo Yoon.

**Validation:** Seung Zhoo Yoon.

**Writing – original draft:** Hyub Huh.

**Writing – review & editing:** Joon Ho Yu, Jisu Hong, Mee Ju Lee, Jang Eun Cho, Choon Hak Lim, Hye Won Lee, Seung Zhoo Yoon.

Seung Zhoo Yoon orcid: 0000-0002-2467-4304.
